# Integrated mRNA and small RNA sequencing reveals microRNA regulatory network associated with internode elongation in sugarcane (*Saccharum officinarum* L.)

**DOI:** 10.1186/s12864-019-6201-4

**Published:** 2019-11-07

**Authors:** Lihang Qiu, Rongfa Chen, Yegeng Fan, Xing Huang, Hanmin Luo, Faqian Xiong, Junxian Liu, Ronghua Zhang, Jingchao Lei, Huiwen Zhou, Jianming Wu, Yangrui Li

**Affiliations:** 1 Sugarcane Research Institute, Guangxi Academy of Agricultural Sciences/Sugarcane Research Center, Chinese Academy of Agricultural Sciences, East Daxue Road 172, Nanning, 530004 Guangxi China; 2 Key Laboratory of Sugarcane Biotechnology and Genetic Improvement (Guangxi), Ministry of Agriculture, and Guangxi Key Laboratory of Sugarcane Genetic Improvement, Nanning, Guangxi China

**Keywords:** Transcriptome, Next-generation sequencing, Zeatin biosynthesis, Nitrogen metabolism, Plant hormone signal transduction

## Abstract

**Background:**

Internode elongation is one of the most important traits in sugarcane because of its relation to crop productivity. Understanding the microRNA (miRNA) and mRNA expression profiles related to sugarcane internode elongation would help develop molecular improvement strategies but they are not yet well-investigated. To identify genes and miRNAs involved in internode elongation, the cDNA and small RNA libraries from the pre-elongation stage (EI), early elongation stage (EII) and rapid elongation stage (EIII) were sequenced and their expression were studied.

**Results:**

Based on the sequencing results, 499,495,518 reads and 80,745 unigenes were identified from stem internodes of sugarcane. The comparisons of EI vs. EII, EI vs. EIII, and EII vs. EIII identified 493, 5035 and 3041 differentially expressed genes, respectively. Further analysis revealed that the differentially expressed genes were enriched in the GO terms oxidoreductase activity and tetrapyrrole binding. KEGG pathway annotation showed significant enrichment in “zeatin biosynthesis”, “nitrogen metabolism” and “plant hormone signal transduction”, which might be participating in internode elongation. miRNA identification showed 241 known miRNAs and 245 novel candidate miRNAs. By pairwise comparison, 11, 42 and 26 differentially expressed miRNAs were identified from EI and EII, EI and EIII, and EII and EIII comparisons, respectively. The target prediction revealed that the genes involved in “zeatin biosynthesis”, “nitrogen metabolism” and “plant hormone signal transduction” pathways are targets of the miRNAs. We found that the known miRNAs miR2592-y, miR1520-x, miR390-x, miR5658-x, miR6169-x and miR8154-x were likely regulators of genes with internode elongation in sugarcane.

**Conclusions:**

The results of this study provided a global view of mRNA and miRNA regulation during sugarcane internode elongation. A genetic network of miRNA-mRNA was identified with miRNA-mediated gene expression as a mechanism in sugarcane internode elongation. Such evidence will be valuable for further investigations of the molecular regulatory mechanisms underpinning sugarcane growth and development.

## Background

Internode elongation is a major feature that affects plant growth, errectness, biomass and ultimately yield [1]. Thus, the genetics and the regulatory mechanisms of internode elongation in crop plants have been extensively investigated. Genetic and environmental factors such as gene expression [2–4], genomic variation [5, 6], hormonal regulation [7, 8], nutrients [9, 10], light [11], water [12] and temperature [13] control internode elongation. Of these regulatory factors, hormonal manipulation is an effective and efficient approach to promote crop growth to promote productivity [14–16].

Complex hormonal mechanisms are associated with internode elongation. For example, auxin [14], gibberellin [17] and brassinosteroids [18] induce internode elongation. In contrast, abscisic acid [19], ethylene [20] and jasmonic acid [21] suppress internode elongation in plants. Further, different species and growing condition add additional complexity to growth and developmental regulation. Understanding sugarcane crop-specific mechanisms of hormonal regulation stem growth would help improve crop productivity. Alternatively, endogenous hormones can be manipulated by genome editing [22, 23] and RNA interference technologies [24, 25]. microRNAs (miRNAs), being an effective RNA interference mechanisms, show the prospect of regulating hormone production and action [26–28]. TIR1 and AFB, part of auxin signaling, are targets of miR393, and the suppressive effects of miR393 on auxin are indicated in *Arabidopsis* [29]. GAMYB, a gene in gibberellin signal pathway, is regulated by miR159 [30]. Also, hormones regulate miRNA expression in plants. For instance, with deep sequencing of abscisic acid-treated tomato (*Solanum lycopersicum*), 269 differentially expressed miRNAs were identified [31].

Development of sequencing technology has facilitated transcriptome studies that provide unprecedented detail about the molecular biological processes in plants [32, 33]. Transcriptome sequencing approaches promise increased understanding of the expression patterns and molecular regulatory mechanisms in gene expression [34]. By transcriptome sequencing, the genes associated with culm elongation in bamboo (*Dendrocalamus sinicus*) were identified [35]. In another study, transcriptome sequencing showed the changes in gene expression via induction of ethephon in maize (*Zea mays*) plants [36]. These studies provide basic information about the functional genes involved in internode elongation. In cotton (*Gossypium hirsutum*), 64 differentially expressed miRNAs were identified during the fiber elongation process [37]. The miRNA profiles during tissue differentiation and growth revealed by small RNA sequencing may provide new insight for epigenetic regulation, which might determine a starting point toward important questions regarding plant growth.

Sugarcane (*Saccharum officinarum* L.) is an economically important crop that is widely planted in tropical and subtropical regions [38]. Sugarcane is used for producing ethanol and raw sugar; thus, this valuable crop is grown around the world [39]. Understanding the genetic control of sugarcane growth, particularly the biological process of internode elongation, would accelerate the industrial development of sugarcane cultivation. Investigation of miRNA-mRNA networks in sugarcane could reinforce further crop gains. Although several studies demonstrate changes in gene expression or miRNAs during internode elongation [40, 41], the present study focused on the integrated analysis of miRNA and mRNA interactions, which should produce an image of the mRNA-miRNA networks that occur between transcriptional and posttranscriptional regulation in this biological process.

To better understand the molecular changes and regulation of gene expression by miRNA, we sequenced mRNA and small RNA libraries from internode tissues at different stages including the pre-elongation stage, early elongation stage and rapid elongation stage. The libraries from these tissues were sequenced by an Illumina Hiseq 4000 platform. By comparing the differential expression, the candidate genes and miRNAs involved in internode elongation were identified. Furthermore, the mRNA and miRNA interaction network was built by target prediction using a bioinformatic approach. These integrated mRNA and small RNA sequencing results provide pioneering evidence for a view of candidate internode elongation-associated miRNAs in sugarcane and may be useful for development of potential functional markers to develop molecular breeding.

## Results

### mRNA expression profiles in different stages from internodes

To understand the molecular mechanism of internode elongation in sugarcane, nine cDNA libraries from the pre-elongation stage (EI), early elongation stage (EII) and rapid elongation stage (EIII) were sequenced. Three biological replicates (individuals) from the stages were included from which a total of 499,495,518 reads were obtained. After filtering, 484,324,322 clean reads were identified (Table 1). All the clean reads were used to perform de novo assembly, which generated 80,745 unigenes. The average length of unigenes was 900 bp, and N50 was 1600 bp.

**Table 1:** Summary of the RNA-Seq Data

Sample	Before Filter Read Number	After Filter Read Number	GC (%)	Q20 (%)	Q30 (%)
EI-1	50,744,342	49,382,978	55.90%	95.85%	90.31%
EI-2	47,767,534	46,374,160	56.12%	95.70%	90.09%
EI-3	63,212,086	61,335,446	55.75%	95.68%	90.07%
EII-1	46,876,844	45,390,562	56.09%	95.56%	89.86%
EII-2	60,658,428	58,791,718	56.70%	95.61%	89.94%
EII-3	68,915,150	66,614,358	61.35%	95.29%	89.27%
EIII-1	53,018,772	51,122,918	55.71%	95.28%	89.35%
EIII-2	54,777,846	53,228,728	56.98%	95.61%	89.71%
EIII-3	53,524,516	52,083,454	55.99%	95.82%	90.26%

**Fig. 1:**
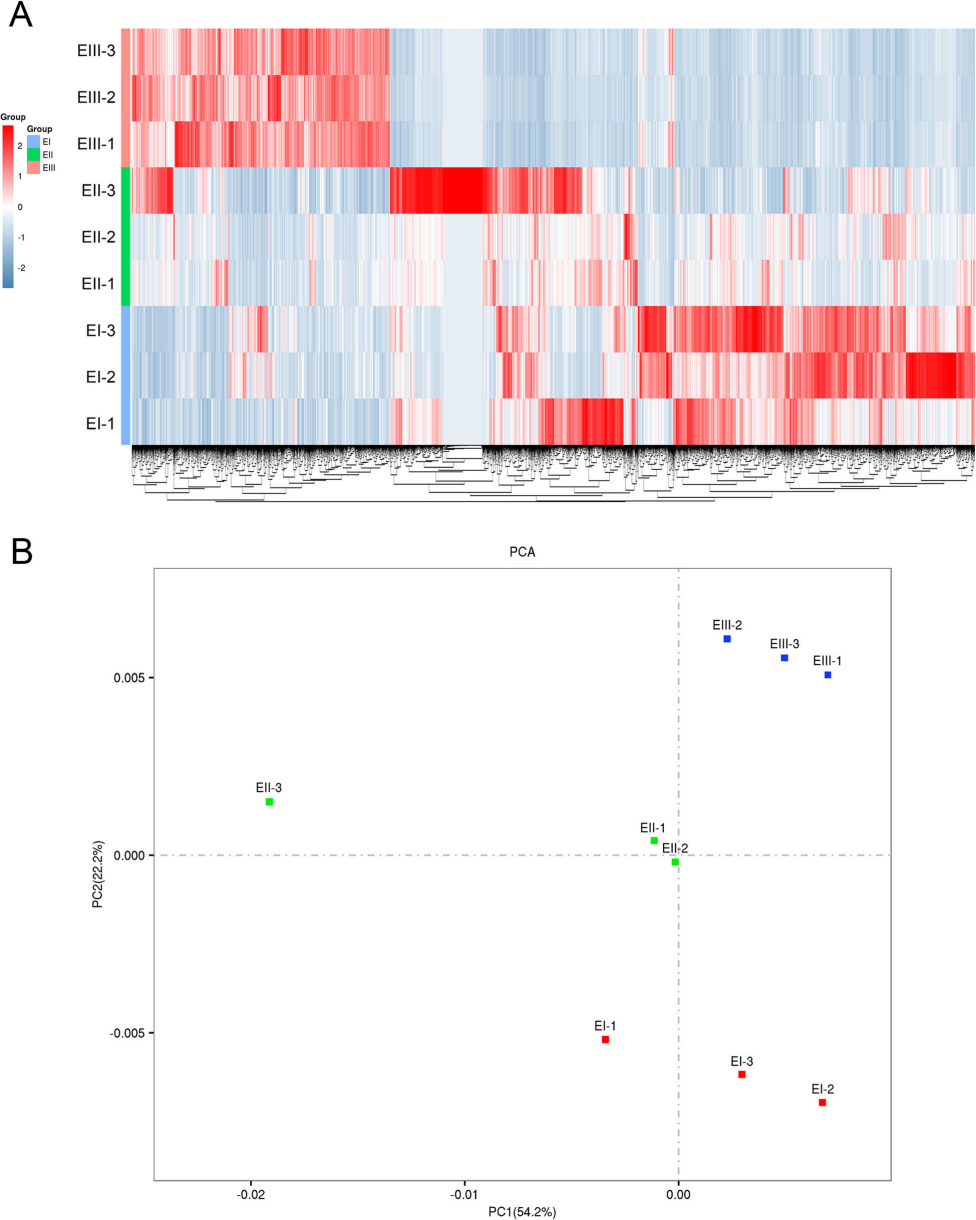
Overview of mRNA Expression Profiles at Different Stages of Internode Elongation in Sugarcane. **a** Heat map comparison of the pre-elongation stage (EI), early elongation stage (EII) and rapid elongation stage (EIII). The Z-score calculated from RPKM values for each gene were used. Gene expression is colored for low (blue) to high (red). Each line represents a single gene and each row shows a library listed on the left. **b** Two-component principal component analysis of the nine transcriptomes from the pre-elongation stage (EI), early elongation stage (EII) and rapid elongation stage (EIII). The percentages represent the variances captured by the principal component 1 (PC1) and principal component 2 (PC2). Red, blue and green dots indicate EI, EII and EIII, respectively

The gene expression in the present study was calculated by the RPKM method. Hierarchical clustering of all the unigenes showed that the biological replicates from each stage were separately clustered together (Fig. 1a). After normalizing the gene expression of all the unigenes, the results of principal component analysis showed a distinct position of the EI, EII and EIII stages, indicating significant changes among the stages in the transcriptome (Additional file 1). There was greater separation on PC1 for samples from the EII groups, indicating a stronger transcriptional differentiation during early elongation stage (Fig. 1b).

### Differentially expressed genes in different stages of internode development

**Fig. 2:**
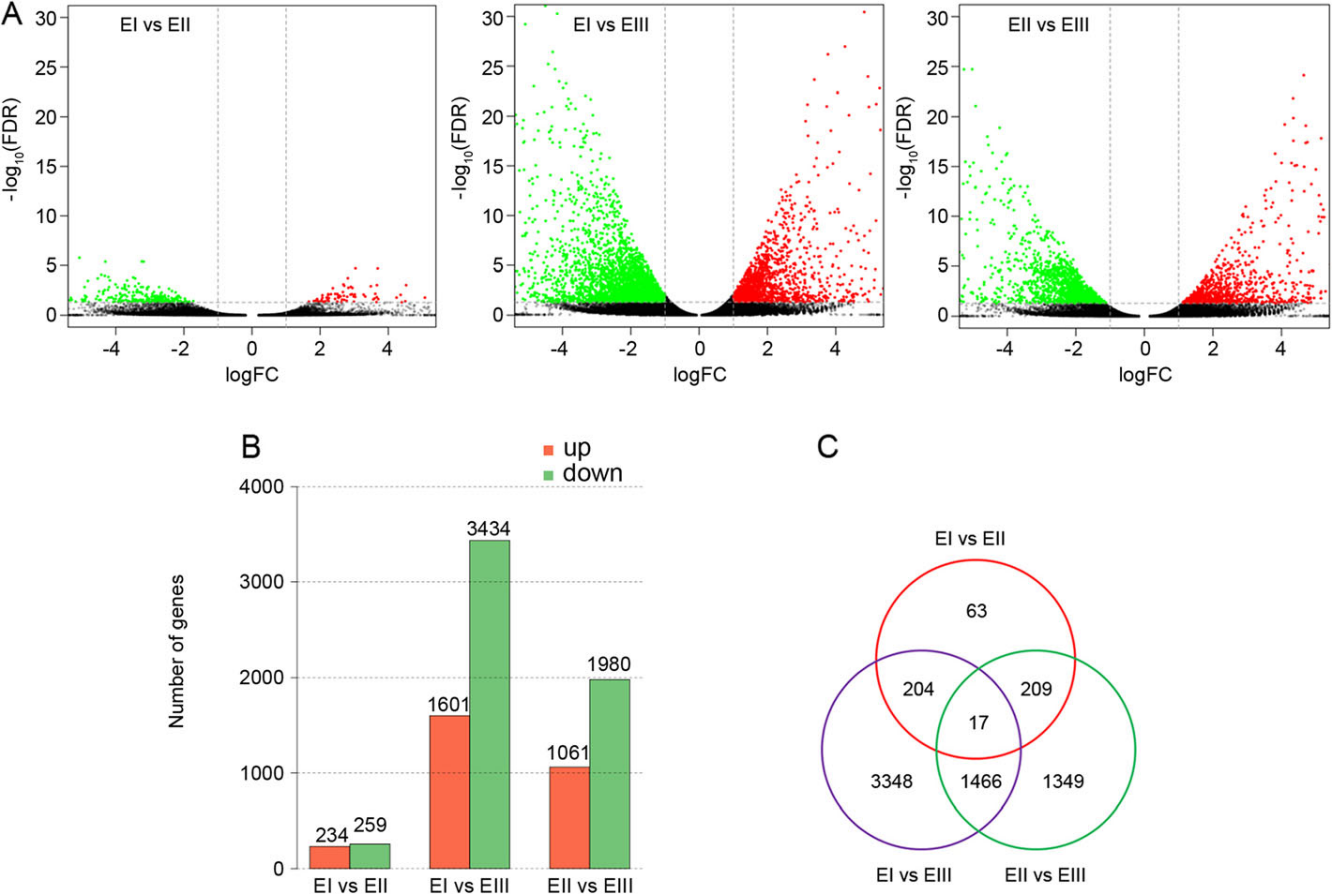
Differential Expression of Genes in Different Stages of Internode Elongation in Sugarcane. **a** Volcano plot shows the differentially expressed genes (green dot: down-regulated genes, red dot: up-regulated genes, black dot: unchanged genes). Each dot represents a single gene. Significant down-regulated genes and up-regulated genes are identified with |log2FC| ≥ 1 and FDR cutoff< 0.05. X axis shows log2FC value and Y axis shows –log_10_ (FDR). **b** Number of differentially expressed genes in the comparisons of EI vs. EII, EI vs. EIII, and EII vs. EIII. Red bars represent the number of up-regulated genes. Green bars represent the number of down-regulated genes. The number on each bar indicates the count of up-regulated or down-regulated genes. **c** Venn diagram shows overlap of differentially expressed genes among the comparisons. The red, purple and green circle represents comparisons of EI vs. EII, EI vs. EIII, and EII vs. EIII, respectively

To compare the gene expression in different stages of internode elongation, the RSEM package was used to identify differentially expressed genes. In the comparisons between EI and EII, EI and EIII, and EII and EIII, 493, 5035 and 3041 differentially expressed genes were identified, respectively (Fig. 2a). Between the EI and EII stages, 234 genes were up-regulated and 259 genes were down-regulated. The most differentially expressed genes were in the comparison between EI and EIII stages, which included 1601 up-regulated and 3434 down-regulated genes at EIII stage. 1,061 and 1,980 genes increased and decreased at EII stage, respectively, by compared with EII and EIII stages (Fig. 2b). The Venn plot showed that 17 differentially expressed genes overlapped among the three comparisons (Fig. 2b, Table 2). The overlap between EI vs. EIII and EII vs. EIII had the maximum number of genes (1,483 differentially expressed genes), whereas the overlap of EI vs. EII and EI vs. EIII had the fewest number of genes (221 differentially expressed genes). The overlap between EI vs. EII and EII vs. EIII had 226 differentially expressed genes (Fig. 2c, Additional file 1).

**Table 2:** Summary of Transcriptome Assembly Statistics

Gene Number	GC (%)	N50 (bp)	Max. length (bp)	Min. length (bp)	Average length (bp)	Total assembled bases (bp)
80,745	50.50	1600	14,063	201	900	72,689,277

### Functional annotation of the differentially expressed genes

**Fig. 3:**
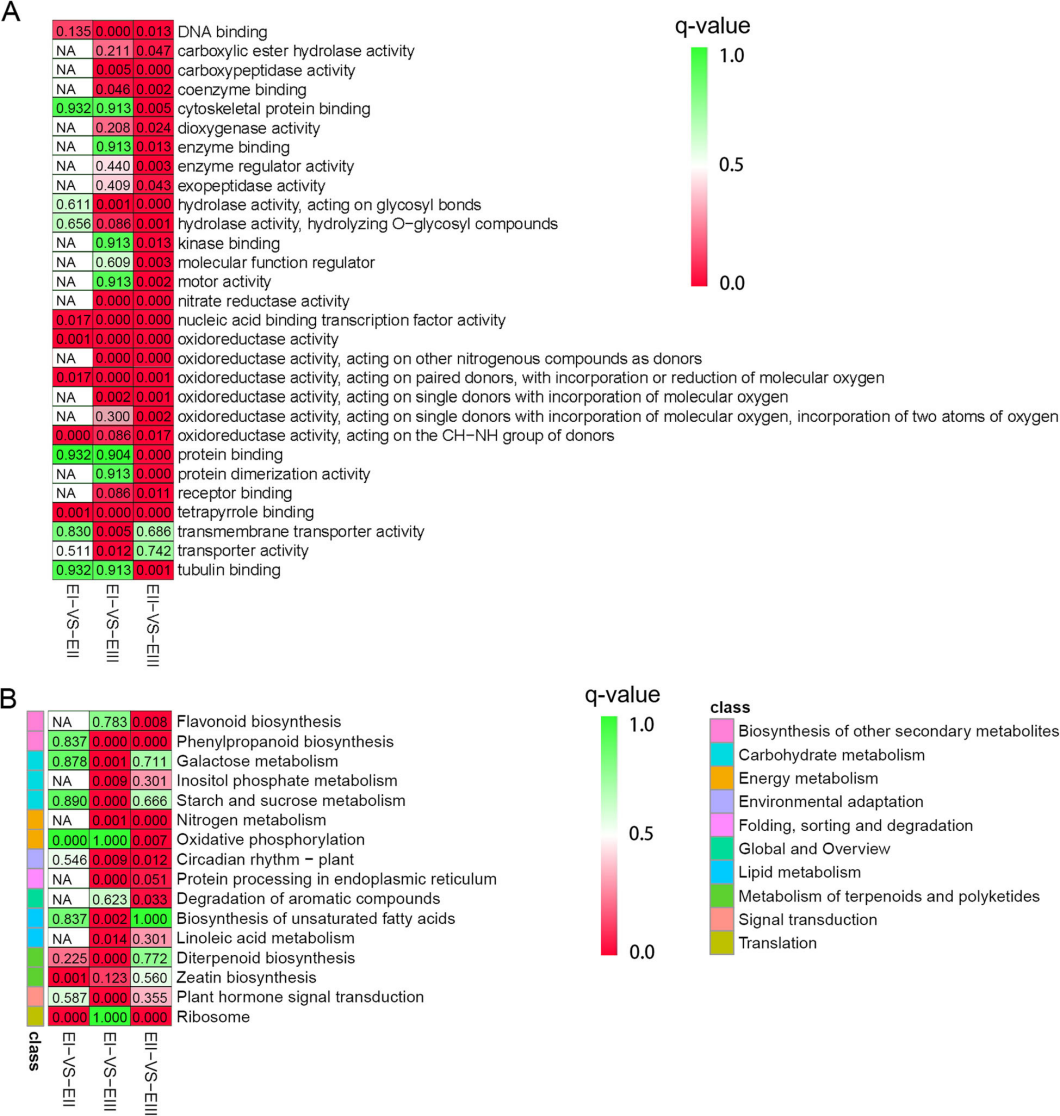
GO (**a**) and KEGG (**b**) Enrichment Analyses of Differentially Expressed Genes in Different Samples. EI, EII and EIII indicate Pre-elongation stage, elongation stage and rapid elongation stage, respectively. q-value is colored for 1.0 (green) to 0.0 (red). NA shows no genes assigned in the category

To reveal the function of differentially expressed genes in different stages from internodes, GO and KEGG enrichment analyses were performed. Only 3 GO terms were enriched (q-value< 0.05) in the comparison EI vs. EII: two oxidoreductase activity terms and tetrapyrrole binding. It was found that 11 GO terms were enriched (q-value< 0.05) in the EI vs. EIII comparison, which included DNA binding, carboxypeptidase activity, hydrolase activity, oxidoreductase activity, nitrate reductase activity, nucleic acid binding transcription factor activity, tetrapyrrole binding, transmembrane transporter activity, and transporter activity. The comparison between EII and EIII indicated that 29 GO terms were enriched (Fig. 3a, Additional file 2).

From the KEGG enrichment results, it was found that 16 KEGG pathways were enriched. From the EI vs. EII comparison, 9 enriched pathways were identified, including “zeatin biosynthesis” and “nitrogen metabolism”, which involved tissue growth. The EI vs. EIII comparison contained 11 enriched pathways, including “plant hormone signal transduction” and “nitrogen metabolism” associated with growth. For EII vs. EIII, 7 enriched pathways were found, including “nitrogen metabolism” (Fig. 3b, Additional file 3).

**Fig. 4:**
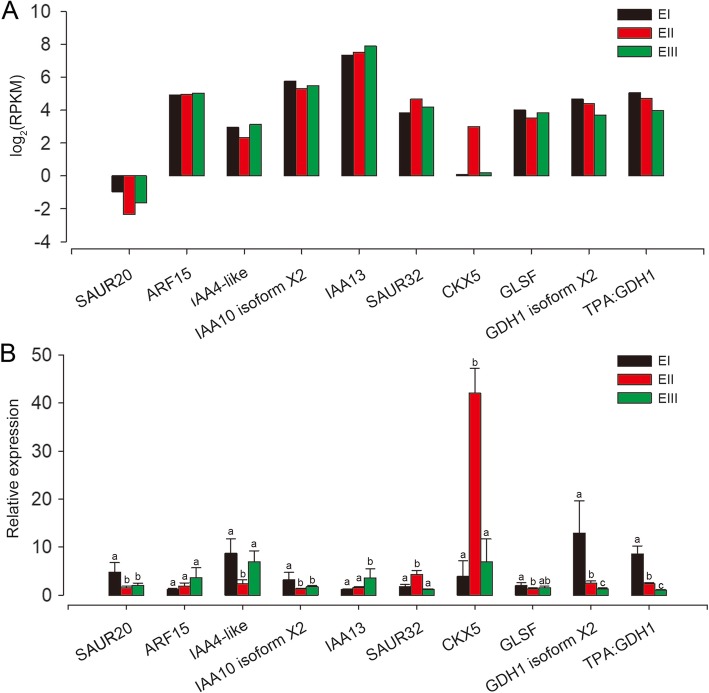
Expression Levels of Ten Candidate Genes in different stages. **a** Expression of 10 candidate genes from RNA-seq calculated by the RPKM method using the RSEM package. The expression is shown as log_2_ (RPKM). **b** Expression of 10 candidate genes from qPCR. The qPCR results are represented at each stage as the mean ± SD. The significant differences (*P* < 0.05) among the PCR results in different groups were indicated using different letters on each bar. EI (black bars), EII (red bars) and EIII (green bars) indicate pre-elongation stage, elongation stage and rapid elongation stage, respectively

The expression profiles of ten candidate genes from “zeatin biosynthesis”, “nitrogen metabolism” and “plant hormone signal transduction” pathways were investigated by qPCR. The expression profiles by qPCR were similar to the results of transcriptome analysis (Fig. 4).

### Sequencing of small RNAs in internodes

The sequencing of small RNAs was investigated to unveil the dynamic regulation of miRNAs on gene expression during internode elongation in sugarcane. Nine small RNA libraries were sequenced, and a total of 137,610,370 clean reads (Table 3) were generated. The length distribution showed that most of these reads were in the range of miRNA from 18 to 24 nt. After removing the rRNA, snRNA, snoRNA, and tRNA by BLAST against the GenBank and Rfam databases, the remaining small RNAs were retained for the following analysis.

**Table 3:** Summary of Small RNA Data and miRNA Annotation

Sample	Read Number	Known miRNA number	Novel miRNA number
EI-1	15,140,390	145	149
EI-2	16,845,194	152	172
EI-3	15,786,890	155	152
EII-1	15,759,580	152	164
EII-2	13,794,648	152	183
EII-3	14,105,373	156	162
EIII-1	14,745,034	157	175
EIII-2	14,864,950	162	170
EIII-3	16,568,311	178	194

### Identification of known and novel miRNAs

The known miRNAs were conducted by blastn to hit the miRBase. A total of 241 known miRNAs were detected in the internode tissues from sugarcane. From all the sequenced libraries, 118 known miRNAs were overlapped in all the groups. miR168-x, miR319-y, miR168-y, miR396-x and miR166-y were the most abundant known miRNAs. The novel miRNAs were predicted by the mireap v0.2 package. A total of 245 novel candidate miRNAs were found in the internodes from sugarcane (Table 3, Additional file 4). Novel-miR0183-5p, novel-miR0209-5p and novel-miR0183-3p were the most abundant novel miRNAs.

### Differentially expressed miRNAs and their targets

**Fig. 5:**
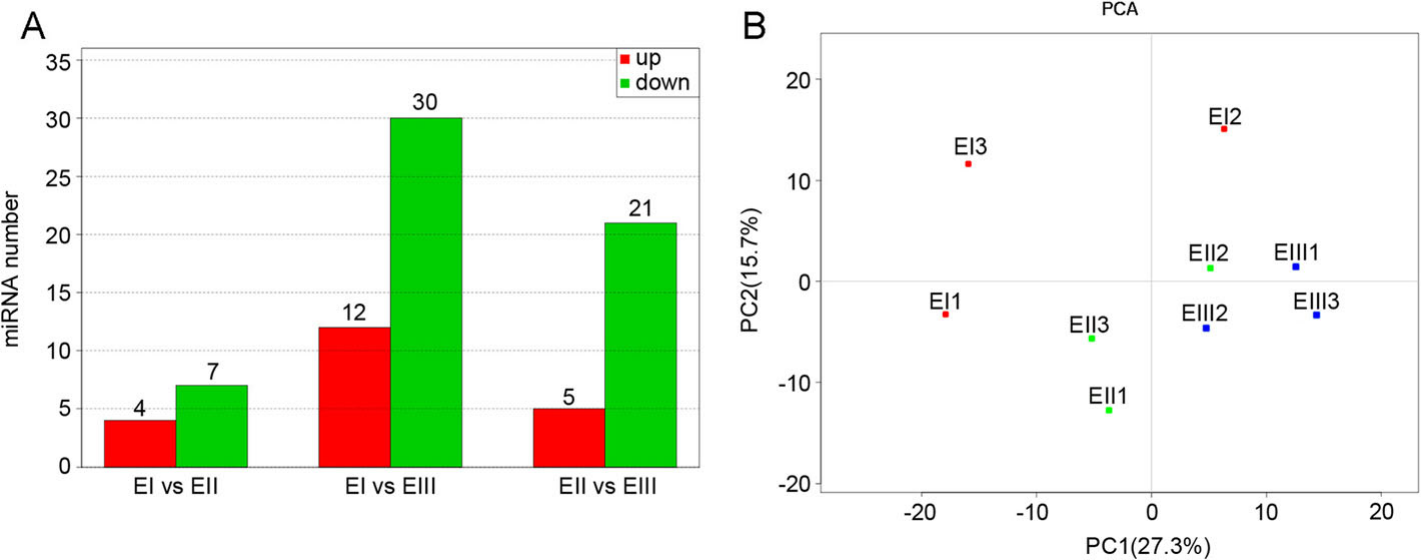
Differential Expression of miRNAs in Different Stages of Internode Elongation in Sugarcane. **a** Number of differentially expressed miRNAs in the comparisons of EI vs. EII, EI vs. EIII, and EII vs. EIII. Red bars represent the number of up-regulated miRNA. Green bars represent the number of down-regulated miRNAs. The number on each bar indicates the count of up-regulated or down-regulated miRNAs. **b** Two-component principal component analysis of the nine miRNA libraries from the pre-elongation stage (EI), early elongation stage (EII) and rapid elongation stage (EIII). The percentages represent the variances captured by the principal component 1 (PC1) and principal component 2 (PC2). Red, blue and green dots showed EI, EII and EIII, respectively

To understand the miRNA regulatory mechanism in internode elongation, the differentially expressed miRNAs were identified by the TPM method. In the comparisons between EI and EII, EI and EIII, and EII and EIII, 11, 42 and 26 differentially expressed miRNAs were found, respectively. Between stages EI and EII, 4 up regulated and 7 down regulated miRNAs were found. The most extensive differentially expressed miRNAs were found between stages EI and EIII, with 12 and 30 that were up-regulated and down-regulated, respectively. Finally, 5 up-regulated and 21 down-regulated miRNAs were found in the comparison EII vs. EIII (Fig. 5a). In the EII vs. EIII comparison, 14 miRNAs with their 75 targets were identified. The principal component analysis indicated a distinct position of the EI, EII and EIII stages (Fig. 5b, Additional file 5).

Targets of the differentially expressed miRNAs were detected. For 8 of the total differentially expressed miRNAs in the EI and EII comparison, the target unigenes were identified (78 in total), whereas no targets were identified for the other 3 miRNAs. In the EI and EIII comparison, 204 targets for 31 miRNAs were identified (Additional file 6).

### Differentially expressed mRNA and miRNA pairs related to internode elongation

**Fig. 6:**
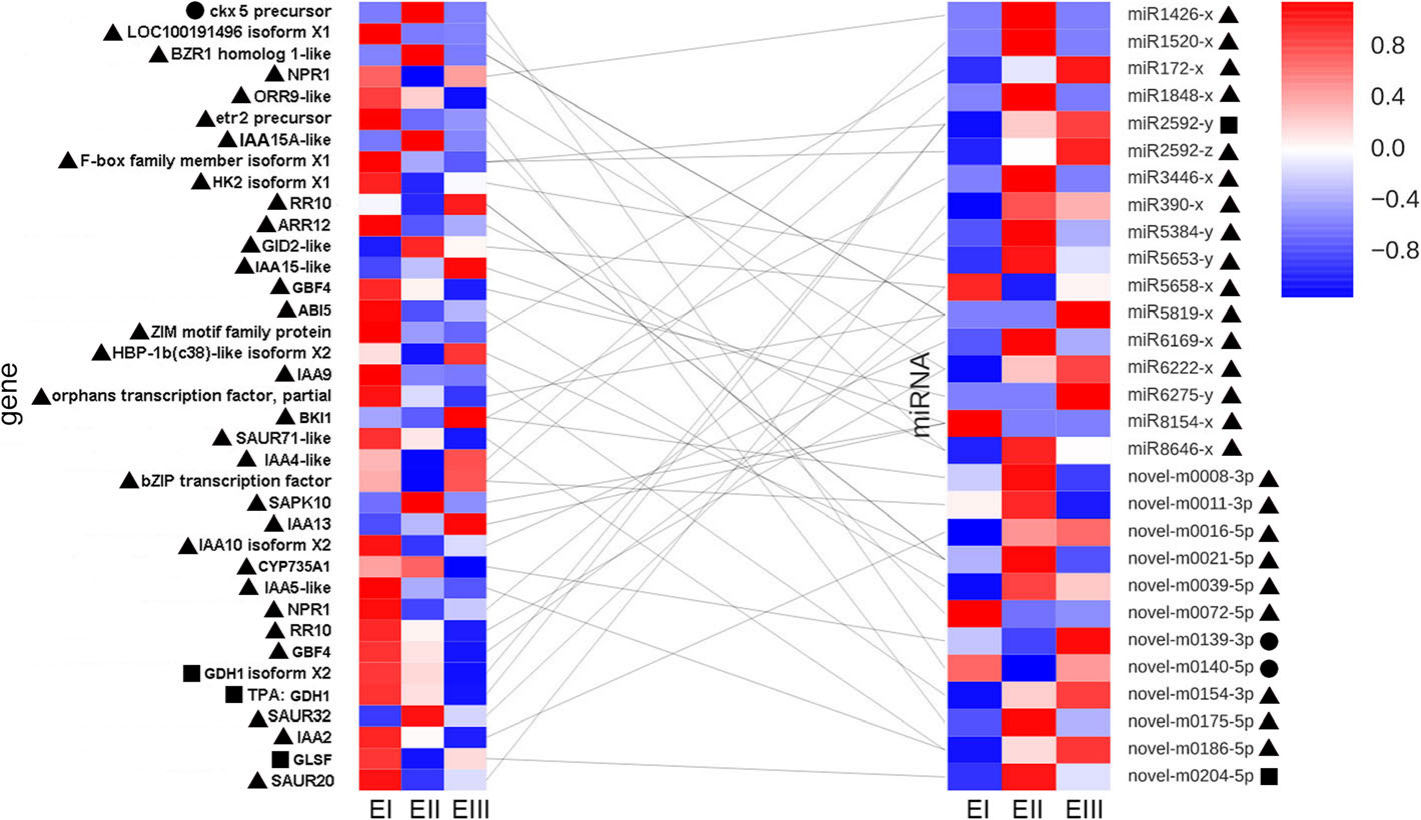
Differentially Expressed miRNAs and their Targets in “Zeatin Biosynthesis”, “Nitrogen Metabolism” and “Plant Hormone Signal Transduction” Pathways. Left: Heat map of miRNA targeted genes; Right: Heat map of the targets of miRNAs. Gene expression is colored for low (blue) to high (red) by e Z-score calculated from RPKM values. miRNA expression is colored for low (blue) to high (red) by e Z-score calculated from TPM values. The left panel: each line represents a library listed below and each row represents a single gene. The left panel: each line represents a library listed below and each row represents a single miRNA. EI, EII and EIII indicate Pre-elongation stage, elongation stage and rapid elongation stage, respectively. Circular, square and triangle indicate genes and miRNAs in “zeatin biosynthesis”, “nitrogen metabolism” and “plant hormone signal transduction” pathways, respectively. Lines between gene and miRNA show the predicted interaction

Internode elongation is a tissue growth process, and therefore, the mRNA and miRNA network related to tissue growth were identified. As found in the present analysis, “zeatin biosynthesis”, “nitrogen metabolism” and “plant hormone signal transduction” pathways were involved in internode elongation. For identification of mRNAs and their corresponding miRNAs in these pathways, 2, 3 and 37 pairs of miRNA-mRNA were found from “zeatin biosynthesis”, “nitrogen metabolism” and “plant hormone signal transduction” pathways, respectively (Additional file 7). Negative correlations (rho between − 0.87 and − 1) between these miRNA and mRNA pairs were observed. The expression of these genes and miRNAs was shown in Fig. 6. In the “zeatin biosynthesis” pathway, novel-m0140-5p and novel-m0139-3p targeted cytokinin dehydrogenase 5 precursor (CKX5) and cytokinin hydroxylase-like isoform X1 (CYP735A1), respectively. miR2592-y targeted glutamate dehydrogenase (GDH1) isoform X2 and TPA, and glutamic dehydrogenase1 (GDH1) and novel-m0204-5p targeted ferredoxin-dependent glutamate synthase (GLSF), the chloroplastic precursor in “nitrogen metabolism” pathways. “Plant hormone signal transduction” had the most miRNA and mRNA pairs. Among all miRNA and mRNA pairs in “plant hormone signal transduction” pathways, 11 genes associated with auxin were found under regulation by miRNAs.

**Fig. 7:**
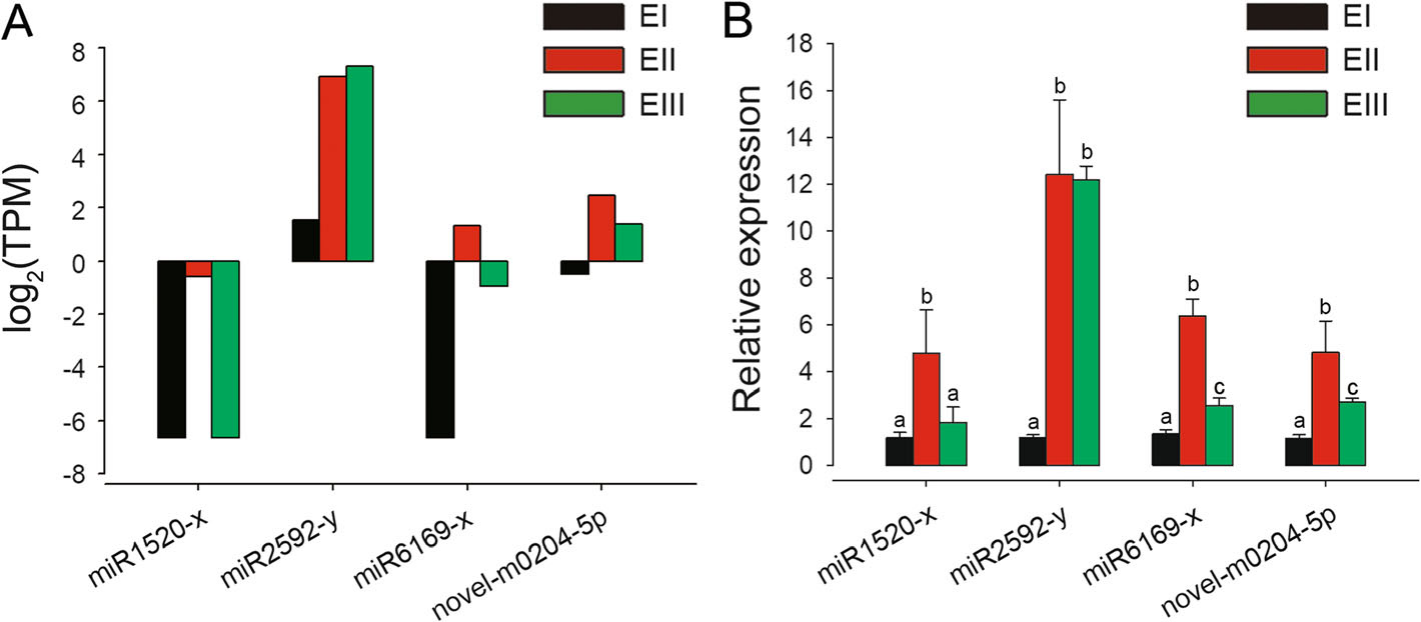
Expression Levels of Four Candidate miRNAs in different stages. **a** Expression of four candidate miRNAs from RNA-seq calculated by the TPM method. The expression is shown as log_2_ (TPM). **b** Expression of four candidate miRNAs from qPCR. The qPCR results are presented at each stage as the mean ± SD. The significant differences (*P* < 0.05) among the PCR results in different groups were indicated using different letters on each bar. EI (black bars), EII (red bars) and EIII (green bars) indicate pre-elongation stage, elongation stage and rapid elongation stage, respectively

To confirm the results of small RNA-seq, qPCR was used to analyze miR2592-y, novel-m0204-5p, miR1520-x and miR6169-x. The expression profiles from qPCR were consistent with the results of the sequencing analysis (Fig. 7).

## Discussion

Stem is the primary valuable tissue for sugarcane, which grows within the internode elongation process. To understand the miRNA-mRNA network during this important process, an integrated mRNA and small RNA sequencing study was done using a next-generation sequencing approach for different internode elongation stages in sugarcane in the present study. This pioneer study provided a molecular basis underlying the elongated internodes in posttranscription regulation.

To investigate the gene profiles in a wide range during sugarcane internode elongation, next-generation sequencing technology was used to analyze the transcriptome changes in the stems from the pre-elongation stage, early elongation stage and rapid elongation stage. The de novo assembly generated 80,745 unigenes. The number of differentially expressed genes in the EI vs. EII, EI vs. EIII and EII vs. EIII comparisons were 493, 5035 and 3041, respectively. Compared with the differentially expressed genes, a significantly higher ratio of unigenes showed similar expression among all the groups, strongly suggesting that the differentially expressed genes were involved in sugarcane internode elongation.

The enriched GO terms identified from the differentially expressed genes were within our expectations. Oxidoreductase activity and tetrapyrrole binding were highly enriched in EI compared with those in the other two groups, indicating the function of these genes in sugarcane internode elongation. Contributing to oxidoreductase activity, flavonoid 3-monooxygenase was significantly higher in the EI pre-elongation stage than in the other groups. Flavonoid 3-monooxygenase is responsible for catalyzing flavonoid to 3′-hydroxyflavonoid. Flavonoids affect plant resistance [42], and flavonoid accumulation may inhibit lignin synthesis [43]. Tetrapyrrole plays various roles in biological processes in plants, including plant growth [44]. For the tetrapyrrole binding term, higher expression of ent-kaurenoic acid oxidase 1 was identified at EII and EIII than EI. This enzyme catalyzes the gibberellin biosynthesis pathway and regulates lignin synthesis [45].

To understand the key pathways in sugarcane internode elongation, the enriched KEGG pathways with differentially expressed genes were identified, and “zeatin biosynthesis”, “nitrogen metabolism” and “plant hormone signal transduction” pathways were enriched with differentially expressed genes. The differentially expressed genes in “zeatin biosynthesis” pathways included CKX3, CKX5, CKX9, CKX10 and CKO2 at EII and EIII compared with EI. It was noted that with the exception of CKX5 at EII, all the other CKX and CKO were significantly down-regulated at EII and EIII stages. CKX/CKO is responsible for degradation of cytokinin [46, 47]. Low expression of the genes in these pathways might be related to high growth activities at EII and EIII stages during internode elongation via cytokinin accumulation [48]. Glutamate dehydrogenase and nitrate reductase were the two enzyme families in “nitrogen metabolism” pathways that might be involved in internode elongation. Transgenic studies in plants found that glutamate dehydrogenase is important for plant growth and productivity [49]. Nitrate as a nutrient for plant growth is regulated by nitrate reductase, indicating changes in nutrient requirements during sugarcane internode elongation [50]. The changing of “plant hormone signal transduction” during internode elongation was also expected. Auxin-related genes including auxin-responsive proteins were decreased at EII and EIII compared with those at EI. Auxin-responsive proteins affect auxin expression by acting on its promoter [51, 52], leading to regulation of sugar accumulation, ethylene biosynthesis and promoting cell elongation.

The observations that several differentially expressed genes were related to internode elongation in the present study indicated that the sugarcane internode elongation was induced by the changes in gene expression in key pathways, suggesting a complex network of the relationships between hormone secretion and internode elongation in sugarcane.

Previous studies demonstrated that plant miRNAs regulate gene expression at posttranscriptional levels related to plant development, including germination, root development, flowering and internode elongation. Here, small RNA sequencing was performed in the stems from the pre-elongation stage, early elongation stage and rapid elongation stage in the present study. A total of 241 known miRNAs and 245 novel candidate miRNAs were identified in these small RNA libraries. Most of these miRNAs were expressed widely among the three different stages. miR168-x, miR319-y, miR168-y, miR396-x and miR166-y were the most abundant known miRNAs, which were consistent with previous miRNA studies in sugarcane [53–55].

Subsequently, the miRNAs involved in control of the gene expression that was related to internode elongation was analyzed. As found in transcriptome analysis, several genes in “zeatin biosynthesis”, “nitrogen metabolism” and “plant hormone signal transduction” pathways participated in sugarcane internode elongation. A total of 43 miRNA-mRNA pairs were found included. Only 6 miRNAs were decreased while other 37 miRNAs increased in EII and EIII stages. Thus, the overall expression trend of miRNAs in these pathways were downregulated and their targeted mRNA were upregulated during elongation process. Among them, CKX5 precursor and CYP735A1 were targeted by two novel miRNAs. This evidence showed that numbers of the candidate miRNA-mRNA remained unidentified to date. Further studies are required to demonstrate the substantive regulatory roles of the novel miRNAs. miR2592 is proposed to participate in the adventitious shoot organogenesis in *Acacia crassicarpa* [56]. In the present study, it was found that miR2592-y was up regulated at EIII and EII stages, whereas its targets were GDH1 isoform X2 and TPA, and GDH1 was down-regulated. In the “plant hormone signal transduction” pathway, 11 miRNA-mRNA pairs were found related to auxin. In these pairs, 5 known miRNAs and 4 novel miRNAs were included. The 5 known miRNAs were miR1520-x, miR390-x, miR5658-x, miR6169-x and miR8154-x. miR1520 is unstable in sugarcane buds under cold stress [57] and only increased at EII stage, which would be expected to lead to an increase in repressing IAA4-like gene expression. The miR390 and its target as auxin response factor were previously reported [58], and therefore, the up-regulation at EII and EIII stages to affect the internode elongation was understandable. miR5658 targeted lateral suppressor-like protein (HaLS-L) and participated in growth and development, as reported previously in carrot [59]. Overexpression of miR8154 in *Taxus* cell lines indicates that miR8154 regulates Taxol, phenylpropanoid, and flavonoid biosynthesis pathways [60]. In this study, high expression of miR8154 at EI stage suggested that the activities of miR8154 were increasing at pre-elongation stage of internode in sugarcane.

**Fig. 8:**
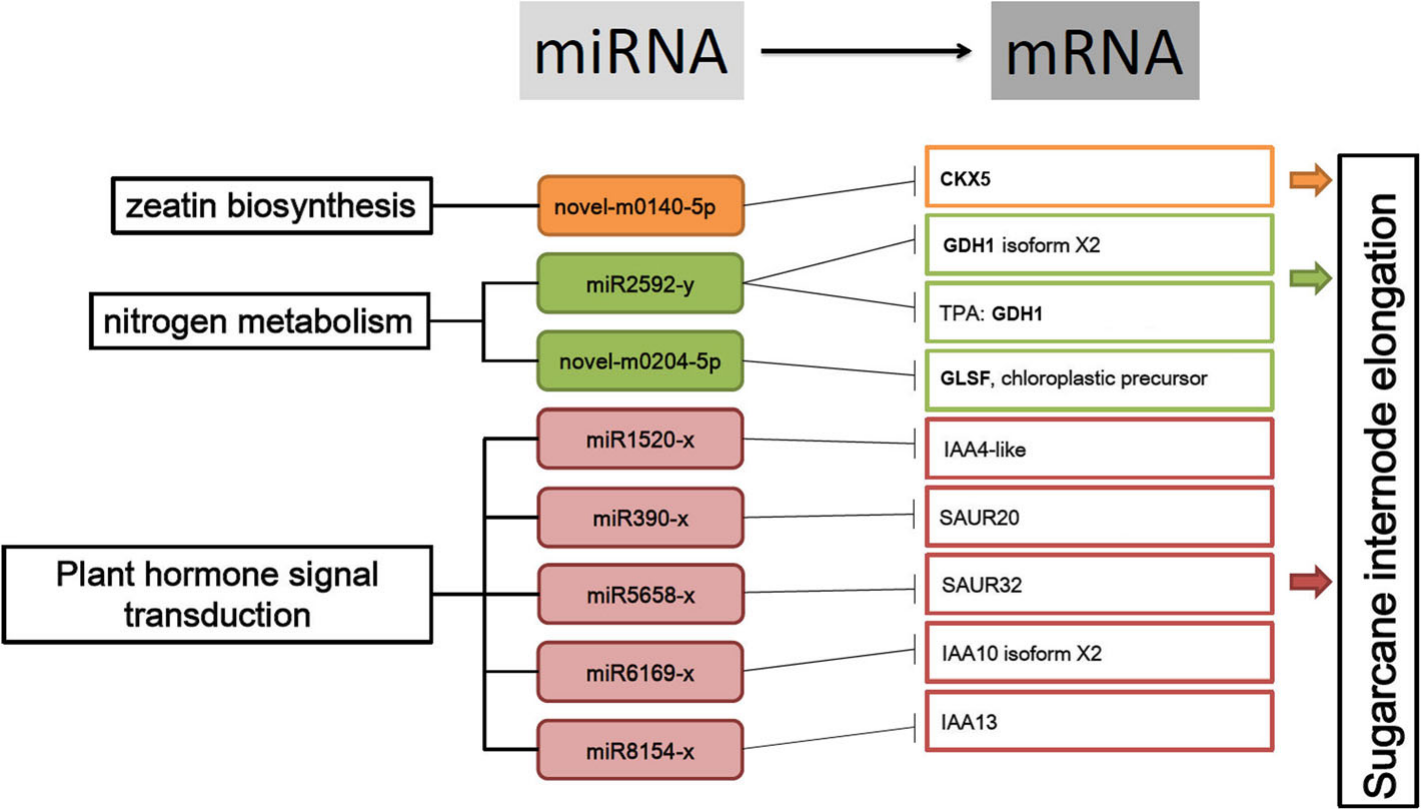
A miRNA-Regulated mRNA Model in Sugarcane Internode Elongation. Novel-m0140-5p was predicted to regulate CKX5 in “zeatin biosynthesis”. In “nitrogen metabolism” pathway, miR2592-y was predicted to regulate GDH1 isoform X2 and TPA: GDH1; novel-m0204-5p was predicted to target CLSF, chloroplastic precursor. miR1520-x, miR390-x, miR5658-x, miR6169-x, and miR8154-x were predicted to target IAA4-like, SAUR32, IAA10 isoform X2 and IAA13, respectively in “plant hormone signal transduction” pathway. These miRNAs targeted genes from “zeatin biosynthesis”, “nitrogen metabolism” and “plant hormone signal transduction” pathways participate in sugarcane internode elongation

With the integrated data from mRNA and small RNA sequencing at different stages of internode elongation, a comprehensive vision could be developed for understanding the miRNA function in this important biological process of sugarcane. Several differentially expressed genes involved in internode elongation related to “zeatin biosynthesis”, “nitrogen metabolism” and “plant hormone signal transduction” pathways were identified. The miRNA data were then used to predict the target mRNA and putative mRNA-miRNA interactions that might participate in internode elongation were found (Fig. 8). Therefore, the present findings demonstrated that miRNAs mediated gene expression in sugarcane internode elongation, which indicates a regulatory network between miRNAs and genes at first glance.

## Conclusions

In summary, mRNA and small RNA profiles were first revealed in sugarcane internode elongation, and comprehensive analysis was performed to identify the regulatory network during this biological process. The results suggested that potential miRNA-mRNA pairs involved in “zeatin biosynthesis”, “nitrogen metabolism” and “plant hormone signal transduction” pathways controlled stem growth and development. This evidence provides valuable information for further functional characterization of the miRNAs and their targets in sugarcane internode elongation.

## Methods

### Plant cultivation and tissue collection

The sugarcane variety GT42, which was bred through sexual hybridization [61], was used as the material in the present study. The sugarcane was grown in an intelligent greenhouse at the Sugarcane Research Institute of Guangxi Academy of Agricultural Sciences, Nanning, China. The bud body from the upper middle stem was selected on March 15, 2016. The seedcane was cut into single-bud setts, and cultivated in a sand table. When the seedlings grew to a height of 8–10 cm, those with consistent size were transplanted to an intelligent greenhouse. After two more month’s growth, when 9–10 true leaves emerged, the plants entered the pre-elongation stage (EI), and the tissue of the stem internode covered by the second true leaf was collected. When 12–13 true leaves emerged, the plants entered the early elongation stage, and the tissue from the stem internode covered by the second true leaf was collected (EII). The rapid elongation stage was determined when 15–16 true leaves emerged, and the stem internode tissue covered by the second true leaf was collected (EIII). Three biological replicates (individuals) were included in the present study for each stage. These tissues were stored at − 80 °C after liquid nitrogen treatment.

### Construction of RNA and small RNA sequencing libraries

According to the supplier’s instructions, total RNA was extracted with RNA Trizol (Invitrogen, Carlsbad, CA, USA). The RNA integrity and quantity were detected by an Agilent 2100 bioanalyzer (Agilent, Santa Clara, CA, USA). Nine mRNA-seq libraries were constructed. First, the mRNAs were enriched by oligo (dT) beads and fragmented into short fragments by fragmentation buffer; second, the first-strand cDNA was reversely transcribed by random primers followed by synthesis of the second-strand cDNA using DNA polymerase I and RNase H; finally, the cDNA fragments were purified with a Qiaquick PCR extraction kit (Qiagen, Hilden, Germany) and ligated with Illumina sequencing adapters (Illumina, San Diego, CA, USA). The mRNA-seq libraries were prepared using pair-end methods (read length was 150 bp) and sequenced using a HiSeq™ 4000 (Illumina, San Diego, CA, USA).

Small RNAs from total RNAs were isolated with 3.5% agarose gel electrophoresis by isolating the 18–30 nt fragments. After ligation with 3′ adapters, the small RNAs were extracted by denaturing urea polyacrylamide gel electrophoresis by isolating the 36–44 nt desired band. Then, 5′ adapters were ligated with the isolated small RNAs following reverse transcription PCR, and the products were isolated again using 3.5% agarose gel electrophoresis at 62–75 bp. The small RNA libraries were sequenced using a HiSeq™ 2000 (Illumina, San Diego, CA, USA).

### Raw data processing and de novo assembly of mRNA-seq reads

Raw reads were stored as fastq and filtered to obtain high-quality clean reads. Briefly, the adapters from raw reads were first removed; then, the reads containing > 10% unknown nucleotides (N) were excluded; and finally, low-quality reads that had 50% low quality (q-value lower than 10) bases were removed. The remaining reads were clean reads.

De novo assembly was conducted using the Trinity program (Version: v2.1.1). The inchworm assembled reads using a K-mer-based approach to generate contigs. Chryalis built a de Bruijn graph for clusters of contigs. Finally, based on de Bruijn graphs, Butterfly analyzed the read pairings from the contigs to generate transcripts and unigenes.

### Identification of differentially expressed genes

Gene expression was calculated based on Reads Per kb per Million reads (RPKM) [62]. The RPKM formula was the following: RPKM = (1 × 10^6^ × C)/(N × L/1000), where C represents the number of reads mapped to the target unigenes, N represents the number of reads mapped to all the unigenes, and L is the length of the target unigenes. The calculation was performed by the RSEM package. The adjustment of the *P*-value for multiple testing by FDR correction was performed for identification of differentially expressed genes with FDR cutoff< 0.05. The differentially expressed genes were identified using the standard as |log2Ratio| ≥ 1 and q < 0.05.

The unigenes from De novo assembly were annotated by four public databases, including the NCBI non-redundant protein database (www.ncbi.nlm.nih.gov), Swiss-Prot protein database (www.expasy.ch/sprot), KOG database (www.ncbi.nlm.nih.gov/KOG) and Kyoto Encyclopedia of Genes and Genomes database (www.genome.jp/kegg), using the Blastx package from NCBI (www.ncbi.nlm.nih.gov/BLAST/). The cutoff e-value was 1e-5, and the best-aligned sequence was determined as the annotation for unigenes. Gene Ontology (GO) was used to reflect the gene function of the large unigene set. The GO annotation of the unigenes was performed using Blast2GO software based on the annotation results from the NCBI non-redundant protein database.

### Processing of small RNA sequencing data

The clean small RNA reads were obtained from raw reads after removing the adapters and low-quality reads (the reads with more than one base with a Q-value lower than 20). Only small RNAs ranging from 18 to 35 nt in length were included in the further analysis. The clean small RNA reads were matched to the GenBank database (www.ncbi.nlm.nih.gov/genbank/, Version 209.0) and Rfam database (rfam.xfam.org/, Version 11) to identify and remove rRNA, scRNA, snRNA and tRNA by blastn (the cutoff e-value was 1e-5).

### Identification of known miRNAs and novel miRNAs

Known miRNAs were identified by hitting the candidate miRNAs miRBase (www.mirbase.org/, Version 21) using blastn (the cutoff e-value was 1e-5). The annotated miRNAs were determined as known miRNAs. Novel miRNAs were predicted by mireap v0.2 (https://sourceforge.net/projects/mireap/). The parameters of mireap were -A 18, −B 25, −a 20, −b 23, −u 20, −e 18, −d 300, −p 16, −v 4, −s 4, and -f 20.

### Prediction of the targets of miRNAs

The software patmatch (ftp://ftp.arabidopsis.org/home/tair/Software/Patmatch/, Version1.2) with default parameters was used to predict the targets of miRNAs. Then, based on the patmatch prediction, the gene-miRNA pairs with Spearman’s correlation coefficient for ranked data lower than − 0.5 were further confirmed the targets of miRNAs. The target gene functions of miRNAs were clarified by GO and KEGG annotation. GO enrichment was calculated by FDR correction, with FDR lower than 0.05 as significantly enriched. The KEGG pathways were performed with FDR correction, with FDR lower than 0.05 as a threshold.

### Identification of differentially expressed miRNAs

The expression of miRNAs was assessed by transcripts per million (TPM), and TPM = miRNA counts/(Total miRNA counts× 10^6^). The fold-changes were calculated using the formula fold-changes = log2(group1/group2). The miRNAs with fold change greater than 2 and *P*-value less than 0.05 were identified as differentially expressed miRNAs.

### Quantitative PCR (qPCR)

Sugarcane tissues were collected as described previously. The total RNA from the different stages EI, EII and EIII was extracted with RNA Trizol (Invitrogen, Carlsbad, CA, USA). Ten of the differentially expressed genes from “zeatin biosynthesis”, “nitrogen metabolism” and “plant hormone signal transduction” pathways were selected for confirmation by qRT-PCR. The primers for qPCR were designed by Primer Express v3.0 (Applied Biosystems, Waltham, MA, USA) (Additional file 8). β-actin was used as the internal control. After RNA extraction, the first-strand cDNA synthesis was obtained using total RNA and a PrimeScript RT Reagent Kit with gDNA Eraser (TaKaRa, Dalian, China) following the manufacturer’s instructions. The cDNA was diluted to 1:20 and used as template for qPCR, which was performed using SYBR®Premix Ex Taq™ II (TliRNaseH Plus) (TakaRa, Dalian, China) according to the manufacturer’s instructions. The PCR was started with initial denaturation at 95 °C for 30 s, followed by 40 amplification cycles at 95 °C for 10 s and 60 °C for 30 s using an ABI 7500 Fast Real Time PCR System (Applied Biosystems, Waltham, MA, USA). The threshold cycle (CT) values were used to calculate relative expression by the 2^-ΔΔCT^ method [63], which was performed using 7500 software version 2.0.4 (Applied Biosystems, Waltham, MA, USA). For qPCR of miRNA, the reverse transcriptase reaction was performed using a microRNA RT kit (TakaRa, Dalian, China). The cycles were the same as with mRNA analysis. All the reactions were performed with three biological repeats. The data were presented as the mean ± SD. The significant differences among the groups were assessed using one-factor analysis of variance (one-way ANOVA) followed by a Bonferroni post hoc test. P-value< 0.05 was considered statistically significant.

## Supplementary information

**Additional file 1.** The RPKM of genes by RNA-Seq.

**Additional file 2.** GO analysis of differentially expressed genes.

**Additional file 3.** KEGG pathway of differentially expressed genes.

**Additional file 4.** Novel miRNAs predicted by mireap v0.2.

**Additional file 5.** miRNA TPM values.

**Additional file 6.** miRNA target predictions.

**Additional file 7.** The differentially expressed mRNA and miRNA pairs in “zeatin biosynthesis”, “nitrogen metabolism” and “plant hormone signal transduction” pathways.

**Additional file 8.** Primers used in this study.

## Data Availability

All the raw data are available in the Sequencing Read Archive (SRA) of NCBI under the BioProject number PRJNA429786.

## Abbreviations

CKX5: Cytokinin dehydrogenase 5 precursor
CT: Threshold cycle
CYP735A1: Cytokinin hydroxylase-like isoform X1
EI: Pre-elongation stage
EII: Early elongation stage
EIII: Rapid elongation stage
FDR: False discovery rate
GDH1: Glutamate dehydrogenase
GLSF: Ferredoxin-dependent glutamate synthase
GO: Gene Ontology
HaLS-L: Lateral suppressor-like protein
KEGG: Kyoto encyclopedia of genes and genomegenomes
kOG: Eukaryotic orthologous group
miRNA: MicroRNA
qPCR: Quantitative PCR
RNA-seq: RNA sequencing or the transcriptome sequencing
RPKM: Reads Per kb per Million reads
rRNA: Ribosomal ribonucleic acid
SD: Standard deviation
snoRNA: Small nucleolar RNA
snRNA: Small nuclear ribonucleic acid
TPM: Transcripts per million
tRNA: Transfer RNA
